# Group II Metabotropic Glutamate Receptors Mediate Presynaptic Inhibition of Excitatory Transmission in Pyramidal Neurons of the Human Cerebral Cortex

**DOI:** 10.3389/fncel.2018.00508

**Published:** 2019-01-08

**Authors:** Marco Bocchio, Istvan P. Lukacs, Richard Stacey, Puneet Plaha, Vasileios Apostolopoulos, Laurent Livermore, Arjune Sen, Olaf Ansorge, Martin J. Gillies, Peter Somogyi, Marco Capogna

**Affiliations:** ^1^Department of Pharmacology, University of Oxford, Oxford, United Kingdom; ^2^Department of Neurosurgery, John Radcliffe Hospital, Oxford University Hospitals NHS Foundation Trust, Oxford, United Kingdom; ^3^Oxford Epilepsy Research Group, NIHR Biomedical Research Centre, Oxford, United Kingdom; ^4^Nuffield Department of Clinical Neurosciences, Medical Sciences Division, University of Oxford, Oxford, United Kingdom; ^5^Department of Biomedicine, Aarhus University, Aarhus, Denmark; ^6^The Danish Research Institute of Translational Neuroscience, Nordic EMBL Partnership for Molecular Medicine – Department of Biomedicine, Aarhus University, Aarhus, Denmark

**Keywords:** presynaptic receptor, glutamatergic, EPSC, cognitive enhancer, transmitter release, epilepsy, mGluR, human cortex

## Abstract

Group II metabotropic glutamate receptor (mGluR) ligands are potential novel drugs for neurological and psychiatric disorders, but little is known about the effects of these compounds at synapses of the human cerebral cortex. Investigating the effects of neuropsychiatric drugs in human brain tissue with preserved synaptic circuits might accelerate the development of more potent and selective pharmacological treatments. We have studied the effects of group II mGluR activation on excitatory synaptic transmission recorded from pyramidal neurons of cortical layers 2–3 in acute slices derived from surgically removed cortical tissue of people with epilepsy or tumors. The application of a selective group II mGluR agonist, LY354740 (0.1–1 μM) inhibited the amplitude and frequency of action potential-dependent spontaneous excitatory postsynaptic currents (sEPSCs). This effect was prevented by the application of a group II/III mGluR antagonist, CPPG (0.1 mM). Furthermore, LY354740 inhibited the frequency, but not the amplitude, of action potential-independent miniature EPSCs (mEPSCs) recorded in pyramidal neurons. Finally, LY354740 did slightly reduce cells’ input resistance without altering the holding current of the neurons recorded in voltage clamp at -90 mV. Our results suggest that group II mGluRs are mainly auto-receptors that inhibit the release of glutamate onto pyramidal neurons in layers 2–3 in the human cerebral cortex, thereby regulating network excitability. We have demonstrated the effect of a group II mGluR ligand at human cortical synapses, revealing mechanisms by which these drugs could exert pro-cognitive effects and treat human neuropsychiatric disorders.

## Introduction

Pre- and postsynaptic metabotropic glutamate receptors (mGluRs) are activated by L-glutamate, the major excitatory transmitter in the mammalian central nervous system (CNS). These receptors regulate neuronal excitability and synaptic plasticity and mediate the actions of neuroactive drugs (Anwyl, 1999; Cartmell and Schoepp, 2002; Niswender and Conn, 2010). Eight subtypes of mGluRs have been identified and these are classified into three groups (mGluRs I, II, and III) according to their amino acid sequence similarities, agonist selectivity, and interactions with transduction mechanisms (Conn and Pin, 1997). Group II receptors (mGlu2 and mGlu3) are intriguing due to their peri- and extra-synaptic location outside the synaptic junction (Shigemoto et al., 1997; Corti et al., 2002), predicting receptor activation in an activity-dependent manner when glutamate spills over from the release site (Scanziani et al., 1997). This process may be of interest not only under physiological but also under pathological conditions (Molinari et al., 2012). Group II mGluRs are often coupled to the cyclic AMP cascade (Conn and Pin, 1997) and are potently activated by a series of compounds developed by several pharmaceutical companies and particularly by Eli Lilly and Company, for example (1)-2-aminobicyclo[3.1.0] hexane-2,6-dicarboxylic acid (LY354740) (Schoepp et al., 1999).

Work performed in rodents shows that the activation of group II mGluRs have pre- and post-synaptic effects, consistent with receptor locations (Anwyl, 1999; Cartmell and Schoepp, 2002; Niswender and Conn, 2010). For example, presynaptic group II mGluRs, expressed by the perforant pathway from the entorhinal cortex depress excitatory synaptic responses recorded from hippocampal CA1 pyramidal cells or interneurons of stratum lacunosum moleculare (Kew et al., 2001; Capogna, 2004; Price et al., 2005). Conversely, activation of postsynaptic mGlu3 expressed by hippocampal CA3 pyramidal neurons enhances excitability by inhibiting a K^+^ conductance and by activating a calcium-sensitive cationic conductance (Ster et al., 2011). Moreover, mGlu3R activation enhances mGlu5R-mediated somatic Ca^2+^ mobilization in pyramidal cells of the mouse prefrontal cortex (Di Menna et al., 2018).

Furthermore, pharmacological or genetic manipulation of mGlu2 gate synaptic plasticity, for instance at hippocampal mossy fiber to CA3 pyramidal cell synapses (Yokoi et al., 1996). Thus, the cellular mechanisms by which group II mGluRs regulate network function are heterogeneous. Additional complexity is given by the fact that group II mGluRs are expressed on axons that target specific cell types but not others (Kintscher et al., 2012).

Group II mGluRs can be down-regulated during development (Doherty et al., 2004), indicating that these receptors could be critical for network maturation as well as for neurodevelopmental disorders. Conversely, group II mGluRs are up-regulated following epileptic seizures (Doherty and Dingledine, 2001). These observations suggest that these receptors could play a role in various brain disorders. Indeed, group II mGluRs are currently investigated as potential drug targets for treating neurological and psychiatric disorders. Several agonists have been developed with the aim of improving cognitive dysfunction in schizophrenia, Alzheimer’s disease and anxiety disorders (Caraci et al., 2018; Ferraguti, 2018; Stansley and Conn, 2018). For example, agonists of group II mGluRs show anxiolytic activity in a wide range of animal models of anxiety disorder (Swanson et al., 2005) and improve cognition (Stansley and Conn, 2018). Experimentally, LY354740 rescues deficits in stereotypy, locomotion, spatial working memory and cortical glutamate efflux induced by pharmacological blockade of NMDA receptors in rodents (Moghaddam and Adams, 1998). Furthermore, several mGlu2 positive allosteric modulators (PAMs) have shown efficacy in preclinical animal models of schizophrenia (Spooren et al., 2000; Galici, 2005) (see review: Maksymetz et al., 2017).

Behavioral effects of mGluR activation in humans provide translational validity of the results obtained in animal models. For example, group II mGluR agonists attenuate NMDA receptor activation-induced deficits in working memory in human subjects (Krystal et al., 2005). However, clinical trials using group II mGluR agonists or PAMs in psychiatric patients have not led to the introduction of novel clinical treatments (see reviews: Maksymetz et al., 2017; Ferraguti, 2018). Reasons for translational failure are complex, including species differences, lack of appropriate animal models, insufficient dosing and limited bioavailability of the drugs tested (Jucker, 2010). As for many other receptor systems, there is a gap between the vast amounts of information available on the actions of group II mGluRs in rodent versus human CNS. The aim of the present study is to help closing this gap and to elucidate the action of the potent and selective group II mGluR agonist LY354740 on pyramidal neurons recorded from acute slices derived from surgically removed human cortical tissue.

## Materials and Methods

### Ethics Statement and Patients

Surgical specimens were obtained from the temporal neocortex of drug resistant temporal lobe epilepsy (TLE) (patients A–E, Table 1A, five females) and from the temporal and frontal neocortex of low grade glioma oncological patients with brain tumors (patients F–H, Table 1B, one female, two males) operated at the John Radcliffe Hospital, Oxford, United Kingdom. All studied tissues were necessarily removed during the surgical procedure and were surplus to diagnostic requirements. Ethics approval was sought and obtained from Research Ethics Committees, National Health Service, Health Research Authority, United Kingdom (NRES Committee South Central – Oxford C: reference 15/SC/0639; NRES Committee East of England – Cambridgeshire and Hertfordshire: reference 14/EE/1098). Fully informed written consent was obtained from each patient who participated.

**Table 1(A):** Clinical data of temporal lobe epilepsy cases.

Patient		Surgery details			Seizure pathology			Histological findings at site of pathology		Medication at time of surgery (dose/day, in mg) - duration			Medication history (dose/day, in mg) - duration			Other relevant pathology
Code	Age^∗^	Sample origin site ^∗^^∗^	Anesthesia	No. of cells	Age at onset	Seizure frequency pre-op	Semiology	Cortical dysplasia	Sclerosis/other histological alterations	Anti-convulsive medication	Psychiatric medication	Other medication^∗∗∗^	Anticonvulsive medication	Psychiatric medication	Other medication^∗∗∗^	Medical history
A	56–60	Right inferior temporal gyrus	Dexamethasone, flucloxacillin, metaraminol, paracetamol, propofol, remifentanil	2	54	Clusters every 2 weeks	Complex and simple partial seizures, no secondary generalization	Inconclusive	Sclerosis ^∗∗∗∗^ – right hippocampus	Phenytoin (500) – 1 year; clobazam (20) – 1 year	Prochlorperazine (15) – 1 year	Betahistine (24) – 1 year; propranolol (30) – 1 year	Carbamazepine (200) – 2 days; levetiracetam (250) – 5 months; pregabalin (25) – 1 month; valproate (1000) – 1 year	Amitriptyline (10) – 9 months	Bendroflumethiazide (2.5) – 9 months	Parieto-occipital meningioma, secondary focal seizures – resected 2005; brainstem cavernoma, telangiectasia, hemorrhage – right pons; brainstem syndrome; temporal choroid plexus meningioma/papilloma – resected 2014
B	26–30	Left middle temporal gyrus	Flucloxacillin, gentamicin, metaraminol, propofol, remifentanil, vecuronium	5	21	1–3 focal seizures per day	Complex partial seizures, occasional secondary generalization	No dysplasia	Diffuse gliosis – left temporal neocortex; ILAE type 1 sclerosis – left hippocampus	Lamotrigine (200) – 3 years; clobazam (20) – 1.5 years; topiramate (250) – 3 years	None – n.a.	None – n.a.	Valproate (500) – 2 weeks; carbamazepine (800) – 3 years; lacosamide (200) – 3 months; levetiracetam (750) – 1 month	None – n.a.	None – n.a.	Depression; migraine
C	30–35	Left middle temporal gyrus	Ceftriaxone, dexamethasone, gentamicin, remifentanil, paracetamol, propofol, vecuronium	2	14	Once every 2–3 weeks	Partial seizures, no secondary generalization	Focal cortical dysplasia	Gliosis – left temporal lobe; sclerosis ^∗∗∗∗^ – left hippocampus	Levetiracetam (2000) – 2.5 years; perampanel (8) – 1.5 years	Citalopram (10) – 2.5 years	Cyclosporin (200) – 2.5 years; methotrexate (12.5/week) – 2.5 years; folic acid (5) – 2.5 years; omeprazole (20) – 2.5 years	Eslicarbazepine (1000) – 2.5 years; clobazam (10) – 4 months; phenytoin (500) – 3 years; carbamazepine (1600) – 3 years; lamotrigine (200) – 3 years; pregabalin (600) – 3 years; lacosamide (200) – 3 years; zonisamide (400) – 2 years; topiramate (25) – 1 year; gabapentin (2700) – 3 years	None – n.a.	None – n.a.	Left temporal lobe epilepsy – left temporal lobectomy 2007; benign paroxysmal positional vertigo; psoriasis; depression
D	36–40	Left inferior temporal gyrus	Flucloxacillin, gentamicin, metaraminol, propofol, remifentanil, vecuronium	7	7	Daily	Complex partial seizures, occasional secondary generalization	No dysplasia	ILAE type 2 sclerosis – left hippocampus	Tegretol retard (1600) – 22 years; topiramate (100) – 2.5 years; clobazam (10) – 5 years; sertraline (200) – 5 years	None – n.a.	Cetirizine (10) – not known	Valproate (1000) – 4 years; lamotrigine (200) – 4 years; levetiracetam (1500) – 4 years; phenobarbitone (not known) – in childhood	None – n.a.	None – n.a.	Depression
E	26–30	Right inferior temporal gyrus	Atracurium, co-amoxiclav, dexamethasone, gentamicin, paracetamol, propofol, remifentanil	2	14	Once per week	Complex partial seizures, occasional generalized tonic–clonic seizures	No dysplasia	ILAE type 1 sclerosis – right hippocampus	Lamotrigine (50) – 8 years; levetiracetam (6000) – 6 years; sertraline (150) – 2 years	None – n.a.	Ovranette (150/30) – 10 years; folic acid (5) – not known	Valproate (500) – 2 years	None – n.a.	None – n.a.	Irritable bowel syndrome; anxiety; asthma

*^∗^Patient in this age range; ^∗∗^sample origin site is from the same hemisphere as the pathology; ^∗∗∗^no steroids; ^∗∗∗∗^ILAE type not determined; ILAE, International League Against Epilepsy.*

**Table 1(B):** Clinical data of glioma cases.

Patient		Surgery details			Tumor pathology and medication^∗∗∗^						Seizure pathology and medication (dose/day, in mg) - duration			Other relevant pathology and medication^∗∗∗∗^ (dose/day, in mg) - duration		
Code	Age^∗^	Sample origin site	Anesthesia	No of cells	Diagnosis	WHO grade	Type	Site of tumor pathology	Cortical infiltration	Increased intracranial pressure/oedema	Seizures	Onset	Anticonvulsive medication	Steroid medication	Other medication	Medical history
F	50–55	Right inferior temporal gyrus	Flucloxacillin, gentamicin, metaraminol, propofol, remifentanil, vecuronium	4	Diffuse astrocytoma, not otherwise specified	II	Wild-type	Right temporal lobe	No	No/no	Partial seizures	1970	Carbamazepine^∗∗^ (200) – 47 years; phenytoin (not known) – 31 years; levetiracetam^∗^**^∗^** (1500) – 16 years	Dexamethasone**^∗^**^∗^ (16) – once before surgery	None – n.a.	Posterior fossa astrocytoma – resected 1970; radiotherapy; traumatic brain injury 1990; left temporal meningioma – resected 2016
G	20–25	Left inferior frontal gyrus	Flucloxacillin, gentamicin, metaraminol, propofol, remifentanil, vecuronium	3	Oligodendroglioma	II	IDH1-H132R mutated	Left frontal lobe	No	No/yes	Partial seizure	Two seizures on 29/10/2017	Levetiracetam^∗^**^∗^** (1000) – 2.5 months	Dexamethasone^∗∗^ (8) – 2.5 months	Omeprazole (20) – not known	None
H	20–25	Right inferior temporal gyrus	Flucloxacillin, gentamicin, mannitol, metaraminol, propofol, remifentanil, vecuronium	5	Anaplastic astrocytoma	III	IDH1-H132R mutated	Right temporal lobe	Yes	Yes**^∗^**^∗∗∗∗^/no	None	n.a.	Levetiracetam^∗^**^∗^** (500) – 1.5 months	Dexamethasone**^∗^**^∗^ (16) – 1.5 months	Lansoprazole (20) – not known	None

*^∗^Patient in this age range; ^∗∗^present at time of surgery; **^∗^**^∗∗^no previous oncological treatment; **^∗^**^∗∗∗^no psychiatric medication; **^∗^**^∗∗∗∗^local increase in pressure, no evidence of generalized increase in intracranial pressure; WHO, World Health Organization; IDH1, isocitrate dehydrogenase 1; diagnosis based on the 2016 WHO classification of CNS tumors.*

### Slice Preparation

A small piece of cortical tissue (size < 1 cm^3^) including all layers and some white matter was isolated using a scalpel, removed and immediately immersed in cutting artificial cerebrospinal fluid (ACSF) saturated with carbogen (95% O_2_/5% CO_2_), at ∼4°C. The solution containing the block of tissue was continuously bubbled with carbogen. Transportation from the operating theater to the laboratory lasted 15–60 min. The block of tissue was placed in a petri dish containing ice-cold cutting ACSF bubbled with carbogen. In some cases, the block of tissue was divided into smaller pieces before transportation. Next, the block of tissue was glued on a platform of a vibratome (Microm HM 650 V, Thermo Fisher Scientific) and cut into slices at a setting of 325 μm thickness in cutting ACSF at 4°C. The block of tissue was oriented in a way to slice perpendicular to the pia and parallel to the apical dendrites of the pyramidal cells. The cutting ACSF contained the following compounds (in mM): 65 sucrose, 85 NaCl, 25 NaHCO_3_, 2.5 KCl, 1.25 NaH_2_PO_4_, 0.5 CaCl_2_, 7 MgCl_2_, 10 glucose (pH 7.3, ∼300 mOsm/L). Slices were incubated in cutting ACSF at 36°C. After ∼10 min, the cutting ACSF was replaced with recording ACSF at 36°C using a peristaltic pump (Gilson) operated at ∼5 mL/min. The recording ACSF contained the following compounds (in mM): 126 NaCl, 2.5 KCl, 1.2 NaH_2_PO_4_, 26 NaHCO_3_, 2 CaCl_2_, 2 MgCl_2_, 10 glucose, (pH 7.3, ∼300 mOsm/L). Slices were heated at 36°C for 30 min, after which they were stored at room temperature and continuously bubbled with carbogen.

### Electrophysiological Recordings

Slices were submerged in a recording chamber and stabilized with a plastic string harp. The chamber was continuously perfused with oxygenated recording ACSF at a rate of 10 mL/min by a peristaltic pump (Gilson) at a temperature of 33 ± 1°C. Neurons were visualized using a differential interference contrast (DIC) microscope (Olympus BX51WI) using a LUMPlanFL 60× water objective (Olympus) and equipped with a camera (Zyla, ANDOR) connected to a desktop computer. Glass electrodes (4–6 MΩ) were prepared from borosilicate glass capillaries (1.2 mm; GC120F, Harvard Apparatus) using a DMZ Universal puller (Zeitz-Instrument). Electrodes were filled with an intracellular solution composed of the following (in mM): 126 K-gluconate, 4 KCl, 4 ATP-Mg, 0.3 GTP-Na_2_, 10 Na_2_-phosphocreatine, 10 HEPES, and 0.05% biocytin, with osmolarity of 270–280 mOsmol/L without biocytin, at pH 7.3 adjusted with KOH. Somatic whole-cell patch-clamp recordings were performed from visualized neurons in cortical layers 2–3. Electrophysiological signals were amplified using an EPC10 triple patch clamp amplifier (HEKA Electronik), digitized at 20 kHz for recordings in voltage-clamp mode and at 5 kHz for recordings in current-clamp mode, and acquired using Patchmaster software (HEKA Electronik). All reported voltage values were compensated for a calculated 16 mV liquid junction potential between the ACSF and the recording pipette. Spontaneous or miniature excitatory postsynaptic currents (sEPSCs or mEPSCs) were recorded in continuous voltage clamp with a holding potential of –90 mV (corresponding to the calculated Cl^-^ reversal potential). The mEPSCs were recorded after the addition of 1 μM tetrodotoxin (TTX) to the recording ACSF. Uncompensated series resistance was monitored using a 20 ms-long -10 mV voltage step applied at the beginning of every sweep (i.e., every minute). The sEPSCs or mEPSCs were recorded continuously for up to ∼30 min. After 3–4 min of baseline recording 0.1–1 μM LY354740 was perfused into the recording chamber for 5 min. The drug was subsequently washed out for 10–20 min.

### Signal Analysis and Inclusion Criteria

Analysis of synaptic currents and intrinsic membrane responses was performed using Igor Pro (WaveMetrics). PatchMaster files were loaded into Igor Pro with Patcher’s Power Tools (Max-Planck-Institute, Department of Membrane Biophysics). The input resistance (*R*_in_) was calculated from the slope of steady-state voltage responses to a series of 8–10 subthreshold current injections lasting 400 ms. Spike threshold, half-width and fast after-hyperpolarization (AHP_fast_, in mV) were determined from the first spike in response to a juxtathreshold positive current injection. The spike half-width was defined as the duration at half-amplitude measured between the threshold potential and the peak of the action potential. The membrane time constant τ was estimated from the monoexponential curve fitting of voltage responses to a -30 pA hyperpolarizing pulse. The membrane capacitance was calculated as the ratio between membrane τ and *R*_in_. The rheobase (in pA) was determined as a 50 ms current injection, able to generate a spike in 50% of the cases in 10 trials. The instantaneous firing rate (in Hz) was defined as the number of action potentials evoked during a 1 s-long depolarizing current pulse of twice the amplitude of the rheobase current. The adaptation index (range, 0–1) was defined as the ratio between the first and last inter-spike intervals (ISIs; in ms) elicited by the same current pulse used to measure the instantaneous firing rate. The resting membrane potential was estimated by averaging a 20 s current-clamp trace recorded at a 0 pA holding current. Changes in *R*_in_ evoked by LY354740 application were assessed in voltage clamp mode by applying regular hyperpolarizing voltage steps (-10 mV). For these experiments, *R*_in_ was calculated as follows:

$$R_{\mathrm{in}}=R_{\mathrm{tot}}-R_{s}$$

where *R*_tot_ is the total resistance and *R*_s_ is the series resistance. *R*_s_ was calculated as follows:

$$R_{s}=\frac{V}{I_{P}}$$

where *V* is the amplitude of the voltage step and *I*_p_ is the amplitude of the peak of the capacitive current transient. *R*_tot_ was calculated as follows:

$$R_{\mathrm{tot}}=\frac{V}{I_{s}}$$

where *I*_s_ is the amplitude of the steady state current.

The following criteria were determined in order to accept or reject event files: (1) recorded neurons were able to generate at least one spike upon current injection in current clamp mode at the beginning of the recording; (2) the holding current to clamp the cell at -90 mV was <-100 pA; (3) the series resistance was <30 MΩ and did not change more than 20% during the recording. Spontaneous synaptic events were detected using TaroTools toolbox for Igor Pro^1^. The threshold for event detection was set between -5 and -7 pA depending on the signal-to-noise ratio of the recording. Events with 20–80% rise-time longer than 3 ms and half-width shorter than 1 ms were removed. Subsequently, all events were visually inspected for the entire recording period in order to confirm the reliability of the automatic detection. Events were rejected if they did not display typical fast EPSC kinetic (i.e., ratio between decay time and 20–80% rise-time <3). Statistical analysis was performed using Prism software (GraphPad, San Diego, CA, United States), and the tests used are specified throughout the results. Unless indicated otherwise, values presented in the text and in the figures represent the median and the interquartile range (IQR). All data sets were tested for statistically significant outliers using the Rout test (*Q* = 1%).

### Visualization of Recorded Cells

After recording, slices were immersed in a fixative containing 4% paraformaldehyde, 15% v/v picric acid dissolved in 0.1 M phosphate buffer (PB) pH 7.2–7.4, overnight at 4°C. Slices were then thoroughly washed in 0.1M PB until all fixative was removed from the tissue and were either embedded in 20% gelatin and re-sectioned into 60 μm thickness on a vibratome (VT 1000S, Leica) or were processed further without re-sectioning. After permeabilization with Tris Buffered Saline (TBS), containing 0.3% w/v Triton (Tx) (Sigma), the recorded cells were visualized with overnight incubation in Alexa-488-conjugated streptavidin (Invitrogen, Thermo Fisher Scientific), diluted 1:1000 in the same buffer. Sections were then washed with TBS-Tx three times for 10 min and were mounted in Vectashield (Vector Laboratories) on glass slides for microscopic examination. Visualized neurons were examined using an epifluorescent microscope (Leitz DMRB, Leica) equipped with a camera (ORCA-ER, Hamamatsu) and connected to a desktop computer, using a 480/40 excitation and a 527/30 emission filter, corresponding to the fluorophore used. All visualized cells were confirmed to be pyramidal neurons based on the distribution of dendrites and axon and the presence of dendritic spines. Images of Z-stacks of some of the labeled neurons were made using a laser-scanning microscope (LSM 710, Zeiss) with a Plan-Apochromat 20x/0.8 objective (Zeiss).

### Chemicals and Drugs

Salts used in the preparation of the internal recording solution and ACSF were obtained from either BDH or Sigma-Aldrich. LY354740 (1)-2-aminobicyclo[3.1.0] hexane-2,6-dicarboxylic acid, CPPG (*RS*)-α-cyclopropyl-4-phosphonophenylglycine and tetrodotoxin (TTX) were purchased from Tocris Bioscience, LY354740 was stored as frozen aliquots of 10 mM in DMSO. CPPG was stored as frozen aliquots of 100 mM in 1 M NaOH. TTX was stored as frozen aliquots of 1 μM in 10 mM sodium citrate buffer, pH 4.8.

## Results

### Identity and Spiking Patterns of the Recorded Neurons

**FIGURE 1:**
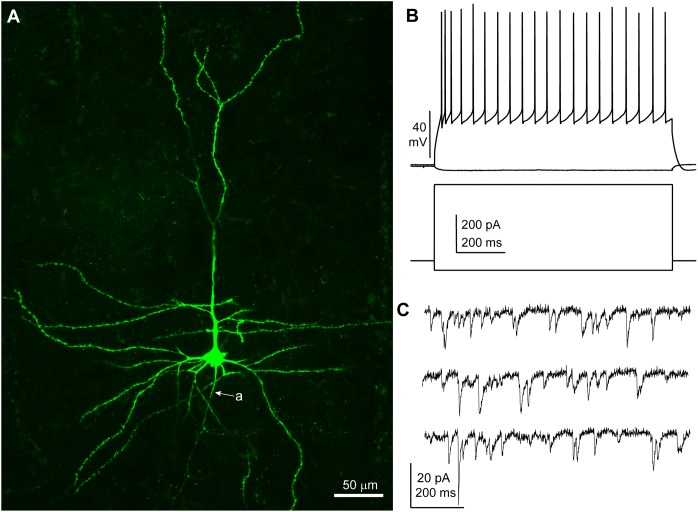
Features of a human cortical pyramidal cell. **(A)** Confocal microscopic image of a biocytin-filled human cortical pyramidal cell in layer 3 (patient/cell code: H26) showing spiny dendrites, a prominent apical dendrite and axon (a) descending toward the white matter; maximum intensity projection of a z-stack of ∼24 μm thickness; 1.8 μm optical slice thickness; interval 0.9 μm; 25 slices). **(B)** Voltage responses of the cell shown in **(A)** recorded in current clamp mode to hyperpolarizing (–50 pA) and depolarizing (+400 pA) current steps (holding potential: –80 mV). **(C)** Representative traces of spontaneous EPSCs recorded in voltage clamp mode at –90 mV; traces are low-pass filtered at 1 kHz and notch filtered at 50 Hz (width: 0.05 Hz). **(A–C)** Same cell.

Neurons were recorded under visual control (*n* = 30; *n* = 8 patients: five TLE, three tumor) and I/V protocols were performed in whole cell current clamp mode to assess the spiking patterns of the recorded cells in response to depolarizing rectangular current pulses. All neurons included in this study displayed membrane responses, action potential kinetics and discharge patterns consistent with features commonly observed in human cortical pyramidal neurons in layers 2–3 (Molnár et al., 2008) (Table 2 and Figure 1). A subset of slices (16 out 30 from six patients, patient IDs: A–D and G, H) were histologically processed to visualize the biocytin-filled neurons. In 15/16 processed slices, the recorded neuron could be identified as pyramidal cells on the basis of spiny dendrites and axonal distribution, consistent with the electrophysiological properties (Figure 1). In one slice the recorded neuron was not recovered.

**Table 2:** Electrophysiological parameters of human cortical pyramidal cells and experimental protocols related to patient and cell codes.

Parameter/experiment	Median	IQR	Cell #	Patient/cell codes
*V*_m rest_ (mV)	-91.6	-89.3 – -93	*n* = 10	A1, B4–B6, C7, D8–D12
*R*_in_ (MΩ)	160.3	123 – 195.5	*n* = 11	A1, B4–B6, C7, D8–D13
Membrane tau (ms)	22.6	19.2 – 28	*n* = 11	A1, B4–B6, C7, D8–D13
Capacitance (pF)	145.7	137.9 – 188	*n* = 11	A1, B4–B6, C7, D8–D13
Rheobase current (pA)	85	39 – 154	*n* = 11	A1, B4–B6, C7, D8–D13
Spike threshold (mV)	-55.8	-48.5 – -61.3	*n* = 11	A1, B4–B6, C7, D8–D13
Spike amplitude (mV)	90.4	87.6 – 91.9	*n* = 11	A1, B4–B6, C7, D8–D13
Spike half-width (ms)	1.1	1 – 1.2	*n* = 11	A1, B4–B6, C7, D8–D13
AHP_fast_ (mV)	12.7	7.2 – 14.5	*n* = 11	A1, B4–B6, C7, D8–D13
Sag ratio (-100 pA)	0.882	0.864 – 0.904	*n* = 9	A1, B5, C7, D8–D13
Rebound amplitude (-100 pA; mV)	1.9	1.3 – 3.5	*n* = 9	A1, B5, C7, D8–D13
Instantaneous firing rate (Hz)	14	12 – 17.5	*n* = 11	A1, B4–B6, C7, D8–D13
Adaptation index	0.233	0.189 – 0.322	*n* = 11	A1, B4–B6, C7, D8–D13
sEPSC frequency (Hz)	2.4	1.4 – 8.2	*n* = 10	A1, B2–B4, C8, D9, D12, E16–E18
sEPSC amplitude (pA)	13.2	8 – 15.6	*n* = 10	A1, B2–B4, C8, D9, D12, E16–E18
mEPSC frequency (Hz)	1.7	1.6 – 3.3	*n* = 9	D13, D14, E15, F19–F21, G23–G25
mEPSC amplitude (pA)	8.6	8.1 – 10.2	*n* = 9	D13, D14, E15, F19–F21, G23–G25
LY354740 (1 μM) tested on sEPSCs	–	–	*n* = 6	A1, B2–B4, C8, E16
LY354740 (0.1 μM) tested on sEPSCs	–	–	*n* = 2	D9, D12
CPPG (0.1 mM) + LY354740 (1 μM) tested on sEPSCs	–	–	*n* = 6	F22, H26–H30
LY354740 (1 μM) tested on mEPSCs	–	–	*n* = 6	F19–F21, G23–G25
LY354740 (0.1 μM) tested on mEPSCs	–	–	*n* = 3	D13, D14, E15
LY354740 (1 μM) tested on holding current	–	–	*n* = 11	A1, B4, C8, E16, E17, F19–F21, G23–G25
LY354740 (0.1 μM) tested on R_in_	–	–	*n* = 5	D9, D12, D13, D14, E15
LY354740 (1 μM) tested on R_in_	–	–	*n* = 6	F19, F20, F21, G23, G24, G25
CPPG (0.1 mM) + LY354740 (1 μM) tested on R_in_	–	–	*n* = 6	F22, H26, H27, H28, H29, H30


*V_*m*__*rest*_, resting membrane potential; *R*_*in*_, input resistance; AHP_*fast*_, fast afterhyperpolarization; sEPSCs, spontaneous excitatory postsynaptic currents; mEPSCs, miniature excitatory postsynaptic currents; IQR, inter-quartile range.*

### Pharmacological Activation of Group II mGluRs Depresses Excitatory Synaptic Transmission in Human Cortical Pyramidal Cells

Based on the evidence of group II mGluR modulation of glutamatergic transmission and pyramidal cell excitability in the rodent brain (Anwyl, 1999), we tested the effect of these receptors in human cortical pyramidal cells. We recorded sEPSCs from pyramidal cells in voltage clamp mode at -90 mV. The median frequency of sEPSCs was 2.4 Hz (IQR: 1.4–8.2 Hz), whereas the median amplitude was 13.2 (IQR: 8–15.6 pA; *n* = 10 from five patients).

**FIGURE 2:**
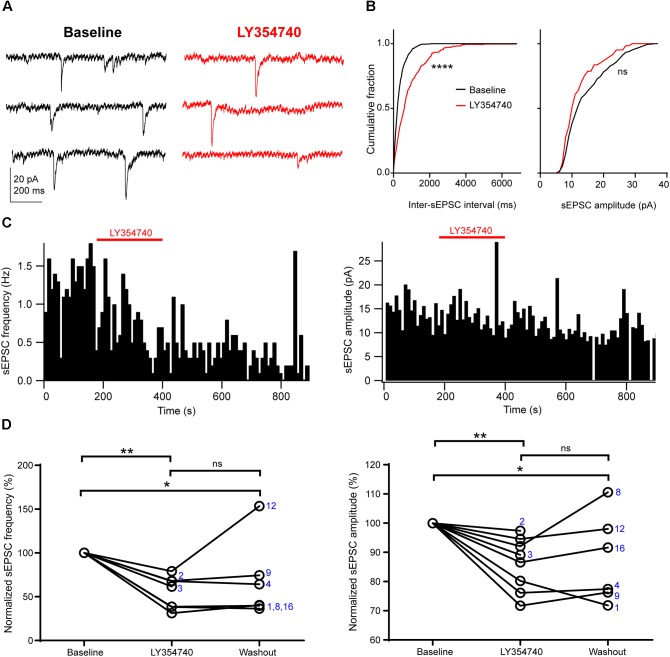
Activation of group II mGluRs depresses excitatory synaptic transmission in human cortical pyramidal cells. **(A)** Representative traces in voltage clamp mode (–90 mV) during baseline and application of the group II mGluR agonist LY354740 (0.1 μM) to a pyramidal cell. **(B)** Cumulative probability distributions of the inter-sEPSC intervals and sEPSC amplitudes for the cell shown in **(A)**. LY354740 significantly reduces sEPSC frequency (left, *p* = 0.0001, Kolmogorov–Smirnov test), and non-significantly decreases sEPSC amplitude (right, *p* = 0.056, Kolmogorov–Smirnov test). **(C)** Event time histograms (bin size: 10 ms, cell in **A,B**) showing the effect of LY354740 application on sEPSC frequency (left) and amplitude (right). **(D)** Baseline normalized effects of LY354740 (0.1 or 1 μM) on individual cells (see Table 2) show significant reduction of sEPSC frequency (left, *p* = 0.003 Kruskal–Wallis test; baseline vs. LY354740 *p* < 0.01, baseline vs. washout *p* < 0.05, LY354740 vs. washout *p* > 0.05, Dunn’s *post hoc* test, *n* = 8) and sEPSC amplitude (right, *p* = 0.003 Kruskal–Wallis test; baseline vs. LY354740 *p* < 0.01, baseline vs. washout *p* < 0.05, LY354740 vs. washout *p* > 0.05, Dunn’s *post hoc* test, *n* = 8). In some cells, washout could not be analyzed due to changes in series resistance (>20% baseline). Numbers denote codes of individual cells (see Table 2). ^∗^*p* < 0.05, ^∗∗^*p* < 0.01, ^∗∗∗∗^*p* < 0.0001.

Application of the selective group II mGluR agonist LY354740 (0.1 μM, *n* = 2; or 1 μM, *n* = 6) significantly depressed the frequency and the amplitude of sEPSCs (*p* = 0.003, Kruskal–Wallis test, pooled *n* = 8, Figure 2). Specifically, LY354740 reduced sEPSC frequency by 35% (median; IQR: 32–61%; *n* = 8) from baseline (*p* < 0.01) and sEPSC amplitude by 12% (median; IQR: 7–21%; *n* = 8) from baseline (*p* < 0.01, Dunn’s *post hoc* test). The depression of sEPSCs partially persisted after 10–20 min post-LY354740 recovery period and could not be fully washed out (baseline vs. washout *p* < 0.05; LY354740 vs. washout *p* > 0.05, LY354740 *n* = 8, washout *n* = 6, Dunn’s *post hoc* test).

**FIGURE 3:**
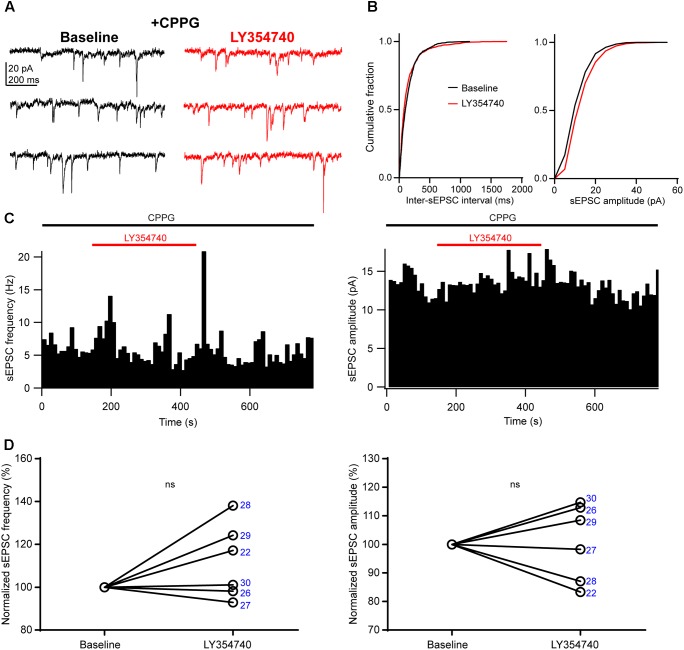
An antagonist of group II/III mGluRs prevents depression of excitatory synaptic transmission by LY354740. **(A)** Representative traces in voltage clamp mode (–90 mV) during baseline and application of LY354740 (1 μM) in the presence of the group II/III mGluR antagonist CPPG (0.1 mM) in a neuron. **(B)** Cumulative probability distributions of the inter-sEPSC intervals and sEPSC amplitudes for the cell shown in **(A)**. LY354740 does not reduce sEPSC frequency (*p* = 0.967, Kolmogorov–Smirnov test) or amplitude (*p* = 0.999, Kolmogorov–Smirnov test). **(C)** Event time histograms (bin size: 10 ms, cell in **A,B**) showing the effect of LY354740 application on sEPSC frequency (left) and amplitude (right) in presence of CPPG. **(D)**, on average, LY354740 (1 μM) does not significantly change sEPSC frequency (left, *p* = 0.312 Wilcoxon test, *n* = 6) or sEPSC amplitude (*p* > 0.999 Wilcoxon test, *n* = 6). Numbers denote codes of individual cells (see Table 2).

We sought to verify that the inhibition of sEPSCs observed upon application of LY354740 was indeed due to group II mGluRs, and not due to other factors, for example spontaneous sEPSC rundown in the slice. To this end, we pre-incubated slices for ∼10 min with the group II/III mGluR antagonist, CPPG (0.1 mM) prior to the additional application of LY354740 (1 μM). Under these conditions, LY354740 did not trigger significant depression of the frequency (change from baseline: median +9%, IQR: -1 to +22%, *p* = 0.312, Wilcoxon test, *n* = 6) or amplitude (change from baseline: median +3%, IQR: -10 to +12%, *p* > 0.999, Wilcoxon test, *n* = 6) of sEPSCs (Figure 3). The frequency and amplitude of sEPSCs in control and in the presence of CPPG were not significantly different (frequency in control: median 2.4 Hz, IQR: 1.4–8.2 Hz, *n* = 10; frequency with CPPG: median 4 Hz, IQR: 1.7–6.5 Hz, *n* = 6, *p* = 0.865, Mann–Whitney test; amplitude in control: median 13.2 pA, IQR: 8–15.6 pA, *n* = 10; amplitude with CPPG: median 10.5, IQR: 9.1–11.7 pA, *n* = 6, *p* = 0.534, Mann–Whitney test), suggesting poor or no endogenous activation of group II mGluRs by glutamate under our experimental conditions.

### Group II mGluRs Depress Excitatory Synaptic Transmission Mainly via a Presynaptic Effect

**FIGURE 4:**
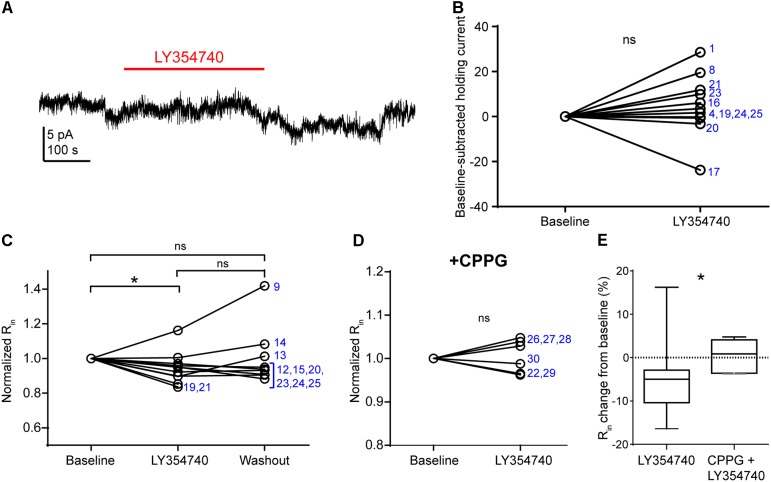
Activation of group II mGluRs does not lead to detectable inward currents in human cortical pyramidal cells. **(A)** Representative trace in voltage clamp mode (–90 mV) during baseline and application of LY354740 (1 μM). The trace was processed with a low pass filter (5 Hz stop band), a notch filter (50 Hz) and boxcar averaging (window of 50 data points; original sampling rate: 20 kHz) to remove synaptic events and isolate the holding current. **(B)** LY354740 (1 μM) does not significantly affect the holding current (*p* = 0.175, Wilcoxon test, *n* = 11). Numbers denote codes of individual cells (see Table 2). **(C)** Baseline normalized effects of LY354740 (0.1 or 1 μM) on individual cells (see Table 2) show significant reduction of *R*_in_ (median change –5%, IQR: 3–10%, *p* = 0.04 Kruskal–Wallis test; baseline vs. LY354740 *p* < 0.05, baseline vs. washout *p* > 0.05, LY354740 vs. washout *p* > 0.05, Dunn’s *post hoc* test, *n* = 11). **(D)** On average, LY354740 (1 μM) does not significantly impact pyramidal cells’ *R*_in_ when CPPG (0.1 mM) is pre-applied (*p* = 0.7, Wilcoxon test, *n* = 6). **(E)** Boxplot showing significantly bigger reduction of *R*_in_ between application of LY354740 only and LY354740 with CPPG (*p* = 0.03, Mann–Whitney test). In some cells, washout could not be analyzed due to changes in series resistance (>20% baseline). Numbers denote codes of individual cells (see Table 2). ^∗^*p* < 0.05.

In pyramidal cells of the rodent hippocampus (Ster et al., 2011) and primate prefrontal cortex (Jin et al., 2017), the activation of group II mGluRs change postsynaptic conductances. The observed depression of sEPSCs in our experiments may be due to: (1) presynaptic inhibition of glutamate release; (2) a hyperpolarization of other pyramidal cells innervating the recorded pyramidal cell; (3) a purely postsynaptic effects on the recorded cells (e.g., changes of AMPA receptor conductance), or a combination of the above. To discriminate between these scenarios, we tested in voltage clamp mode whether LY354740 application altered the holding current and the *R*_in_ of the recorded pyramidal cells (Figure 4). We did not observe a change in the holding current upon the application of LY354740 (1 μM) in neurons at -90 mV (change from baseline: median 2.6 pA, IQR -0.5 to 10.4 pA, *p* = 0.175, Wilcoxon test, *n* = 11, Figures 4A,B), suggesting that, at least at this membrane potential, pyramidal cells are not hyperpolarized by this drug. Nonetheless, LY354740 application (0.1 μM, *n* = 5; or 1 μM, *n* = 6) triggered a small but significant reduction of *R*_in_ (median change -5%, IQR: 3–10%, *p* = 0.04, Kruskal–Wallis test, pooled *n* = 11; baseline vs. LY354740 *p* < 0.05, LY354740 vs. washout *p* > 0.05, baseline vs. washout *p* > 0.05, Dunn’s *post hoc* test). This effect was absent when CPPG (0.1 mM) was applied prior to the additional application of LY354740 (1 μM; *p* = 0.7, Wilcoxon test, *n* = 6). The reduction of *R*_in_ from baseline triggered by LY354740 was significantly smaller in the presence of CPPG (*p* = 0.03, Mann–Whitney test), suggesting that this effect was mediated by activation of group II mGluRs. Thus, group II mGluRs appear to trigger small changes in membrane conductance in pyramidal cells.

**FIGURE 5:**
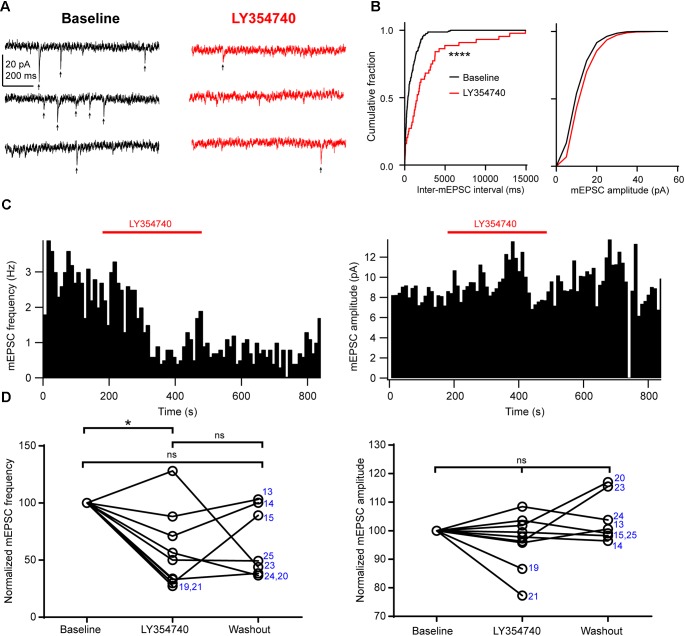
Activation of group II mGluRs depresses excitatory synaptic transmission via a presynaptic effect. **(A)** Representative traces in voltage clamp mode (–90 mV) from a neuron during baseline and application of the group II mGluR agonist LY354740 (1 μM) in the presence of 1 μM tetrodotoxin; mEPSCs are marked with arrows. **(B)** Cumulative probability distributions of the inter-mEPSC intervals and mEPSC amplitudes for the cell shown in **(A)**. LY354740 significantly reduces mEPSC frequency (left, *p* < 0.0001, Kolmogorov–Smirnov test) but not mEPSC amplitude (right, *p* = 0.999, Kolmogorov–Smirnov test). **(C)** Event time histograms (bin size: 10 ms, cell in **A,B**) showing the effect of LY354740 application on mEPSC frequency (left) and amplitude (right). **(D)** Baseline normalized effect of (0.1 μM, *n* = 3 cells, or 1 μM, *n* = 6 cells) on individual cells. LY354740 significantly reduces mEPSC frequency (left, *p* = 0.019 Kruskal–Wallis test; baseline vs. LY354740 *p* < 0.05, baseline vs. washout *p* > 0.05, LY354740 vs. washout *p* > 0.05, Dunn’s *post hoc* test, *n* = 9) but not mEPSC amplitude (right, *p* = 0.239 Kruskal–Wallis test; *n* = 9). In some cells, washout could not be analyzed due to change in series resistance (>20% baseline). Numbers denote codes of individual cells (see Table 2). ^∗^*p* < 0.05, ^∗∗∗∗^*p* < 0.0001.

Next, in order to further discriminate between presynaptic and postsynaptic effects, we tested the drug on spontaneous mEPSCs recorded from pyramidal cells. In the presence of 1 μM TTX to block action potentials, mEPSC frequency had a median value of 1.7 Hz (IQR: 1.6–3.3 Hz) and mEPSC amplitude had a median value of 8.6 pA (IQR: 8.1–10.2 pA, *n* = 9). Application of 0.1 μM (*n* = 3 cells) or 1 μM LY354740 (*n* = 6) caused a significant reduction in the frequency of mEPSCs (change from baseline: median -50%, IQR: -29 to -67%, *p* = 0.019 Kruskal–Wallis test; baseline vs. LY354740 *p* < 0.05, Dunn’s *post hoc* test, *n* = 9 cells pooled, Figure 5). In contrast, mEPSC amplitude was not significantly altered by LY354740 (change from baseline: median -2%, IQR -4 to +2%, *p* = 0.239 Kruskal–Wallis test; *n* = 9, Figure 5). Taken together, these results suggest that activation of group II mGluRs leads to presynaptic and postsynaptic effects in pyramidal cells. However, the lack of changes in holding current (at least at -90 mV) and the modest changes in *R*_in_ suggest that the depression of excitatory transmission is predominantly caused by inhibition of glutamate release from glutamatergic terminals innervating pyramidal cells.

## Discussion

We have demonstrated that activation of group II mGluRs inhibits spontaneous excitatory transmission impinging onto pyramidal neurons in layers 2–3 of the human cerebral cortex. The group II mGluR agonist LY354740 depressed the frequency and amplitude of action potential-dependent sEPSCs but did not alter the holding current of pyramidal cell, although it slightly reduced the cells’ input resistance. It also reduced the frequency but not the amplitude of action potential-independent mEPSCs. Overall, these findings suggest that group II mGluRs act predominantly via a presynaptic effect on glutamate release, rather than via hyperpolarization of layers 2–3 pyramidal cells or postsynaptic effects on ionotropic glutamate receptor conductance. However, we cannot exclude that activation of postsynaptic group II mGluRs in pyramidal cells can lead to network effects. Increases in membrane conductance could lead to membrane hyperpolarization when the cell’s membrane potential is more depolarized and/or to a reduction of the cell’s excitability. If large numbers of pyramidal neurons express group II mGluRs in the human cortex, even a small hyperpolarization or reduction in excitability could alter network dynamics. Our results confirm the negative ‘autoreceptor’ role of this receptor observed in rodent neocortex (e.g., Libri et al., 1997) and hippocampus (e.g., Capogna, 2004). Although group II mGluRs on glutamatergic terminals are activated by glutamate, the source of this transmitter may be either the same terminal that is being suppressed or nearby terminals. In fact, the receptors are widely distributed along the axons and the non-junctional bouton membrane, and glutamate release sites are densely distributed in the neuropil. Therefore, the term ‘autoreceptor’ describes the chemical nature of the terminal and the receptor, and not necessarily a terminal-autonomous regulatory mechanism.

Preclinical studies have demonstrated that group II mGluR agonists exhibit antipsychotic-like properties in animal models of schizophrenia (Stansley and Conn, 2018). However, when these compounds were tested in clinical trials on schizophrenic patients, results were not encouraging (Muguruza et al., 2016). This may be due to patient selection or previous exposure to atypical antipsychotics (Muguruza et al., 2016; Maksymetz et al., 2017). Nonetheless, it is important to acknowledge that rodent animal models do not fully capture the complexities of psychiatric disorders and often show poor predictive power for drug efficacy (Nestler and Hyman, 2010).

Unraveling the cellular effects of group II mGluRs in human cortex could facilitate the identification of causes underlying the poor efficacy of current drugs and help design new, more effective pharmacological treatments. We have tested the effect of a ligand of broad interest for drug development, an agonist for type II mGluRs, on synaptic events recorded from human cortical neurons. Our results demonstrate that this ligand, tested previously in rodents, is effective in inhibiting EPSCs recorded in human pyramidal cells. Future studies using human cortical slices may also help to design novel antipsychotic drugs by shedding light on the physiological effects of PAMs of group II mGluRs (e.g., LY487379 or AZD8529), designed to improve cognition deficits with fewer side effects than more traditional drugs (Singewald et al., 2015).

Our data show that LY354740 inhibited the frequency and amplitude of action potential-dependent sEPSCs as well as the frequency, but not amplitude, of action potential-independent mEPSCs. The latter effect by a drug is usually interpreted as inhibition of spontaneous vesicle fusion and transmitter release (Scanziani et al., 1992). However, LY354740 also appears to trigger postsynaptic effects, as indicated by the small but significant effect of LY354740 on cells’ input resistance. Future studies could confirm the effect of group II mGluRs on neurotransmitter release by examining calcium-dependent release evoked by electrical stimulation of a set of presynaptic fibers. Calcium-dependent and independent neurotransmitter release have been often assumed to share similar mechanisms (Scanziani et al., 1992), although more recent data suggest that the pool of vesicles underlying spontaneous transmitter release can be different from that involved in evoked release (Sara et al., 2005).

In the present study, we could test only a small number of neurons due to limited availability of human cortical tissue. Therefore, we have not explored the subcellular or molecular mechanisms leading to presynaptic depression of glutamatergic transmission. In rodent hippocampus, these receptors are found at pre-terminal axons and on the boutons at some distance from release sites (Shigemoto et al., 1997; Corti et al., 2002), but whether similar localization also occurs in the human cerebral cortex is not known. Based on rodent data, group II mGluR activation could act downstream on N- and P/Q type Ca^2+^ channels, presynaptic K^+^ channels, intrinsic release machinery proteins or could be activated by retrograde release of endogenous transmitters from the postsynaptic cells (Niswender and Conn, 2010). Furthermore, our data cannot rule out a contribution of group II mGluRs expressed by pyramidal cells’ dendritic membrane, perhaps at some distance from the soma. Finally, mGlu3 is expressed in human astrocytes where it enhances the uptake of glutamate from the synapse by increasing the expression of glial glutamate transporters (Aronica et al., 2003), and this mechanism could contribute to the inhibition of glutamatergic synaptic events observed in our experiments.

We have only investigated the activation of group II mGluRs by an exogenous ligand. However, it is unclear whether endogenous ligands (likely glutamate) could also modulate neurotransmission. The frequency and amplitude of sEPSCs detected in the presence of the mGluR II/III antagonist CPPG were similar to control conditions, suggesting undetectable levels of group II mGluR activation by endogenous glutamate in acute slices. Future studies should investigate activity-dependent activation of group II mGluRs, as demonstrated in rodents hippocampus (Kew et al., 2001, 2002; Capogna, 2004), which may also be relevant to mechanisms of synaptic plasticity (Tzounopoulos et al., 1998). In the human cortex, group I mGluRs trigger long-term depression of excitatory transmission impinging on fast-spiking GABAergic interneurons (Szegedi et al., 2016), but it is not yet known whether group II mGluRs can mediate analogous effects.

It is yet to be determined whether group II mGluRs depress transmission at all glutamatergic synapses on pyramidal cells or at specific pathways. Spontaneous synaptic events recorded from pyramidal neurons could be mainly due to glutamate released either from thalamus and/or from cortical inputs (Pasquale and Sherman, 2012). Future experiments using selective stimulation of anatomically identified fibers could determine what input(s) physiologically activate group II mGluRs in the human cerebral cortex.

Whether group II mGluR activation leads to depression of most glutamatergic synapses or to suppression of specific pathways, a marked reduction of excitatory transmission in layers 2–3 pyramidal cells is likely to trigger dramatic network effects. Intriguingly, altered activity of cortical neuronal ensembles has been reported in two mouse models of schizophrenia (Hamm et al., 2017), a finding that suggests that group II mGluRs – despite unsuccessful clinical trials to date – could still represent a promising target for this disorder.

It is likely that activation of group II mGluRs causes changes in neurotransmission that are not restricted to glutamatergic synapses onto pyramidal cells. First, these receptors might be located on glutamatergic axons innervating at least some GABAergic interneurons, similar to the modulation of excitability of fast-spiking GABAergic neurons of human cortex by group I mGluRs (Szegedi et al., 2017). Group II mGluRs might be expressed in a cell-type dependent manner, comparable to the expression of mGluR7 (Shigemoto et al., 1996), or other presynaptic metabotropic receptors, e.g., the cannabinoid 1 receptor (Ludanyi et al., 2008). Second, group II mGluRs might also modulate GABAergic transmission (Hayashi et al., 1993; Ohishi et al., 1994). It will be interesting to investigate whether similar mechanisms occur in the human cortex.

Which group II mGluRs were activated in the present experiments? LY354740 is a potent and selective agonist (up to 1 μM) at mGlu2 and mGlu3 receptors with an EC50 of about 10–50 nM in the rat cortex, hippocampus and striatum, and 10 or 30 nM in cells expressing recombinant mGlu2 or mGlu3, respectively (Schoepp et al., 1999). The concentrations used in this study, therefore, were several fold higher than the EC50, in order to ensure activation of mGluRs throughout the entire depth of the slice. Although the concentrations used in this study still predict selectivity over groups I and III mGluRs (EC50: 300 μM and 100 μM, respectively), we did not discriminate between mGlu2 and mGlu3 activation (e.g., Johnson et al., 2013). Thus, future investigations could attempt to discriminate between mGlu2 and mGlu3, also because understanding the different roles of these two receptors could help to design more selective drugs.

The action of group II mGluRs is regulated by development, network events and epileptic-like events in rodents (Doherty et al., 2004). Future studies using tissue closer to pathological focus could test whether pathological processes can cause functional upregulation of receptors.

It is important to acknowledge that the use of tissue from human cerebral cortex of patients subjected to neurosurgery has some methodological limitations. One of these is that the tissue may have some pathological features that remain undetected. We have used cortical tissue from people with epilepsy refractory to medications or from low grade glioma tumor patients (except one patient that was grade III). We have performed the experiments only on cortical tissue that was located outside the focal epileptic region. Accordingly, we have not observed any epileptic-like signal in the human cortical slices used for our study, such as rhythmic spike bursts in current clamp or rhythmic sEPSC bursts in voltage clamp. In the samples obtained from the periphery of diffuse gliomas as assessed by magnetic resonance imaging, variability may be caused by the degree of glial infiltration. Another possible limitation is the variability due to heterogeneity of cortical areas of provenance, different age and sex of the patients, their individual clinical and pharmacological history. Despite this variability, we observed basal functional parameters that were rather homogeneous across samples and patients and consistency in the effects mediated by group II mGluRs. In addition, cortical tissue removed from remote brain tumor sites has been used as control, non-epileptic tissue in a study investigating cellular activities in human epileptic tissue (Jiang et al., 2012).

## Conclusion

In conclusion, the present study suggests that the activation of group II mGluRs mainly leads to inhibition of glutamate release at synapses on layers 2–3 pyramidal neurons of human cerebral cortex via presynaptic ‘autoreceptors.’ We have established an experimental framework to test the neurophysiological effects of ligands that are relevant to neuropsychiatric conditions in acute slices of human neocortex. Clarifying the mechanisms of action by these ligands has the potential to shed light on their actions in the human brain and bolster the design of more potent and selective drugs.

## Author Contributions

RS, PP, and VA performed neurosurgery. LL, AS, OA, and MHG helped with human tissue collection and regulated procedures. MB, IL, PS, and MC performed the experiments and analyses, and wrote the manuscript with comments from all the authors.

## Conflict of Interest Statement

The authors declare that the research was conducted in the absence of any commercial or financial relationships that could be construed as a potential conflict of interest.

## Footnotes

1 https://sites.google.com/site/tarotoolsregister
